# Exploring *Weissella confusa* W1 and W2 Strains Isolated from Khao-Mahk as Probiotic Candidates: From Phenotypic Traits to Genomic Insights

**DOI:** 10.3390/antibiotics13070604

**Published:** 2024-06-28

**Authors:** Ei Phway Thant, Komwit Surachat, Sarunyou Chusri, Chonticha Romyasamit, Rattanaruji Pomwised, Monwadee Wonglapsuwan, Thunchanok Yaikhan, Sirikan Suwannasin, Kamonnut Singkhamanan

**Affiliations:** 1 Department of Biomedical Sciences and Biomedical Engineering, Faculty of Medicine, Prince of Songkla University, Songkhla 90110, Thailandkomwit.s@psu.ac.th (K.S.); p_rair@hotmail.com (T.Y.); sirikan4036@gmail.com (S.S.); 2 Translational Medicine Research Center, Faculty of Medicine, Prince of Songkla University, Songkhla 90110, Thailand; 3 Division of Infectious Diseases, Department of Internal Medicine, Faculty of Medicine, Prince of Songkla University, Songkhla 90110, Thailand; sarunyouchusri@hotmail.com; 4 Department of Medical Technology, School of Allied Health Sciences, Walailak University, Nakhonsithammarat 80161, Thailand; chonticha.ro@wu.ac.th; 5 Division of Biological Science, Faculty of Science, Prince of Songkla University, Songkhla 90110, Thailand; rattanaruji.p@psu.ac.th (R.P.); monwadee.wo@psu.ac.th (M.W.)

**Keywords:** *Weissella confusa*, probiotic bacteria, EPS, antibacterial activities, whole genome sequencing

## Abstract

Growing interest in probiotics has spurred research into their health benefits for hosts. This study aimed to evaluate the probiotic properties, especially antibacterial activities and the safety of two *Weissella confusa* strains, W1 and W2, isolated from Khao-Mahk by describing their phenotypes and genotypes through phenotypic assays and whole genome sequencing. In vitro experiments demonstrated that both strains exhibited robust survival under gastric and intestinal conditions, such as in the presence of low pH, bile salt, pepsin, and pancreatin, indicating their favorable gut colonization traits. Additionally, both strains showed auto-aggregation and strong adherence to Caco2 cells, with adhesion rates of 86.86 ± 1.94% for W1 and 94.74 ± 2.29% for W2. These high adherence rates may be attributed to the significant exopolysaccharide (EPS) production observed in both strains. Moreover, they exerted remarkable antimicrobial activities against *Stenotrophomonas maltophilia*, *Salmonella enterica* serotype Typhi, *Vibrio cholerae*, and *Acinetobacter baumannii*, along with an absence of hemolytic activities and antibiotic resistance, underscoring their safety for probiotic application. Genomic analysis corroborated these findings, revealing genes related to probiotic traits, including EPS clusters, stress responses, adaptive immunity, and antimicrobial activity. Importantly, no transferable antibiotic-resistance genes or virulence genes were detected. This comprehensive characterization supports the candidacy of W1 and W2 as probiotics, offering substantial potential for promoting health and combating bacterial infections.

## 1. Introduction

Probiotics are living micro-organisms that provide health benefits to the host when consumed in sufficient quantities. They have gained significant interest due to their potential health advantages, leading to heightened interest in various sectors in recent years [1]. These beneficial microorganisms, often sourced from traditional and fermented foods, are integral to the realm of probiotic foods that contain a single or mixed culture of micro-organisms known to enhance intestinal microbial balance and promote consumer health [2,3]. Among such foods, Khao-Mahk, a traditional Thai fermented food, stands out for its attributed health benefits [1,4].

Previous research has isolated several types of beneficial bacteria from different traditional fermented foods [5,6]. Among them, *Weissella* species are classified under the family *Leuconostocaceae*, order *Lactobacillales*, class *Bacilli*, and phylum *Firmicutes* [7,8] and they have been derived from diverse sources, including the intestines, saliva, feces, and vaginas of humans, vegetables, animals, and fermented foods [9,10,11]. Recently, they have attracted attention for their promising health benefits [12]. The *Weissella* strains, particularly *W. cibaria*, are under scrutiny for their strong probiotic properties, including antibacterial activities. The main antibacterial activity comes from antimicrobial substances produced, such as organic acids, hydrogen peroxide (H_2_O_2_), and bacteriocins, in combination with other metabolites. These substances inhibit the growth of harmful bacteria, helping to maintain a healthy balance of gut microbiota and prevent infections [11,12,13].

Moreover, the *Weissella* strains are recognized for their ability to produce exopolysaccharide (EPS). These polysaccharides are increasingly valued for their potential as prebiotics and for their diverse applications in industries, particularly in bakeries and the production of functional beverages [14]. EPS production can also provide self-protection in challenging environments, promote adherence to the intestine, and exhibit antiadhesive properties against pathogenic bacteria, leading to the inhibition of the colonization of pathogenic bacteria to the intestinal lining, thereby decreasing the risk of infection and promoting intestinal well-being [15,16].

In this study, the probiotic properties and safety of *W. confusa* W1 and W2 strains isolated from Khao-Mahk were investigated by highlighting their antibacterial activities and exopolysaccharide production, which were assayed through phenotypic and genomic characterization. Our findings not only contribute to the understanding of the potential probiotic properties of these strains but also lay the groundwork for future applications in the development of functional foods, dietary supplements, and pharmaceuticals aimed at promoting gastrointestinal health and overall well-being.

## 2. Results and Discussion

### 2.1. Bacterial Isolation and Identification

Two strains, W1 and W2, of *Weissella confusa* were isolated from distinct samples of Khao-Mahk obtained from local markets within Songkhla Province, Thailand. Species identification was initially conducted by Matrix-Assisted Laser Desorption/Ionization-Time of Fight (MALDI-TOF) mass spectrometry, which yielded a score value of >2.0, indicating high confidence in the identification. Subsequent confirmation was achieved through short-read sequencing of genomic DNA using the MGISEQ 2000 platform, which unequivocally classified both strains as *W. confusa*.

### 2.2. Phenotypic Assays

#### 2.2.1. Tolerance Tests to Gastric and Intestinal Conditions

**Figure 1:**
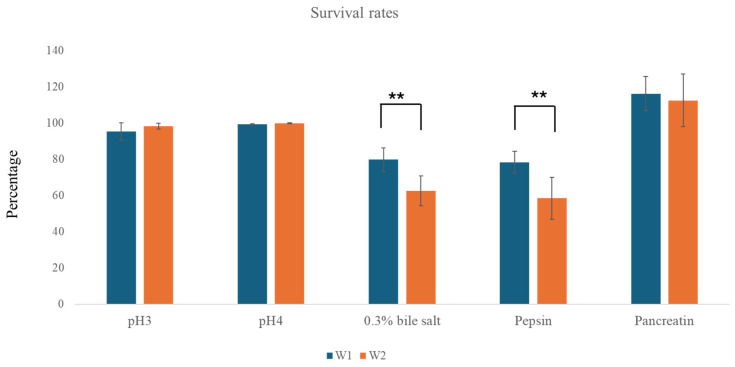
Survival rates of *W. confusa* strains W1 and W2 strains under different gastric and intestinal conditions. Tests were done on three independent experiments. ** indicates the statistically significant difference between W1 and W2 strains (*p* < 0.01).

The tolerance of probiotic strains to the harsh conditions of the gastrointestinal (GI) tract is a crucial factor in their effectiveness as probiotics [11]. In this study, the tolerance of strains W1 and W2 isolated from Khao-Mahk to gastric and intestinal conditions (pH, bile, and enzymes) was evaluated. The mean values of viable cells present in the inoculated MRS at pH 3 and 4 after 3 h of inoculation were found to be over 99% for both strains. Furthermore, both strains exhibited high tolerance to 0.3% bile salts, with survival rates of 79.8% for W1 and 62.5% for W2. The average survival rate of both strains in conditions with gastric enzyme pepsin was over 50%, and both strains were able to grow well in conditions with pancreatin enzymes, which simulate the conditions of the small intestine. However, the survival rates of W1 in conditions with 0.3% bile and pepsin were significantly higher than that of W2 (*p* value < 0.01). The results are shown in Figure 1.

Compared to well-known probiotic strain *Lactobacillus rhamnosus* GG, which is known for its strong tolerance to GI conditions, including extreme acid (up to pH 1.5) [17], *W. confusa* strains in this study exhibited no resistance at that extreme pH, with growth over 99% only up to pH 3. Prior research has demonstrated that tolerances to GI conditions vary among species, and while *Weissella* species can grow only up to pH 3, they are still capable of being used as probiotics [14,18,19]. Growth rates of *W. confusa* strains W1 and W2 in other conditions can be comparable with these common probiotic strains. According to Sadeghi et al. [18], survival levels can be classified into four groups: susceptible (survival rate less than 10%), moderate resistance (between 10% and 60%), good resistance (between 60% and 80%), and very good resistance (greater than 80%). Therefore, both strains showed very good resistance to pH 3, pH 4, and pancreatin, while strain W1 demonstrated higher survivability in the presence of bile and pepsin compared to W2. The observed tolerance to harsh GI conditions could be attributed to the production of stress-response proteins, such as chaperones and proteases, might help maintain cellular functions under stress conditions such as acid and bile stress [20,21].

#### 2.2.2. Auto-Aggregation and Adherence to Caco2 Intestinal Cell Line

**Figure 2:**
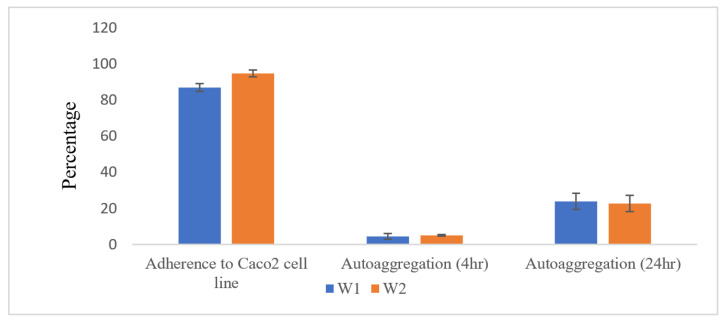
Percentage of bacterial cell attachment to Caco2 cell line and auto-aggregation. Tests were done on three independent experiments.

The auto-aggregation and adherence rates to the Caco2 cell line are displayed in Figure 2. The auto-aggregation rates of strains W1 and W2 were approximately 5% after 4 h of incubation, increasing up to 24% and 22.7% after 24 h, respectively. These findings are concordant with those of Nami Y et al. [14], where the auto-aggregation of *W. confusa* strains ranged from 3.54 ± 0.42% to 33.45 ± 0.36%. Aggregation is a critical probiotic trait as it can enhance their colonization and persistence in the gut, potentially leading to improved health benefits [16].

Regarding adherence to intestinal cells, *W. confusa* W2 exhibited a notably higher adhesion rate (94.74% ± 2.29) compared to *W. confusa* W1, which showed a slightly lower adhesion rate of 86.86% ± 1.94. However, this difference was not statistically significant. This strong adherence to intestinal cells is a characteristic often associated with probiotic strains, indicating their potential to colonize and outcompete pathogenic bacteria within the gut environment [16]. In comparison to previous studies, where *W. confusa* strains displayed lower adhesion rates, our strains W1 and W2 demonstrated significantly enhanced adherence capabilities. For instance, Ayeni et al. [10] reported relatively low adhesion rates for *W. confusa* stains, whereas Nami et al. [14] observed varying adherence capacities among *W. confusa* strains, with strain ABRIIFBI-96 recording the highest adherence rate at approximately 22.59%. These findings suggest strains W1 and W2 possess superior abilities to colonize the intestinal epithelium and interact with the host, thereby enhancing their probiotic potential.

#### 2.2.3. Exopolysaccharide (EPS) Production

**Figure 3:**
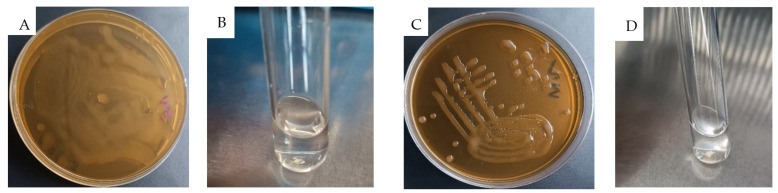
Exopolysaccharide production on 2% sucrose MRS agar and confirmation test in ethanol (**A**,**B**) W1 and (**C**,**D**) W2.

Following a 24 h incubation period, strains W1 and W2 displayed mucoid colonies on MRS plates supplemented with 2% sucrose. Following ethanol treatment of these colonies, aggregation was observed, which is an indication of EPS production, as illustrated in Figure 3. EPS are long-chain sugar polymers produced by certain bacteria, serving various functional roles such as an adherence to intestinal cells, protection against environmental stresses, biofilm formation, and modulation of host–microbe interactions [14,22]. The observed aggregation following ethanol treatment further supports the presence of EPS, as it can create a matrix-like structure conducive to bacterial aggregation [23]. Our findings are in line with previous studies highlighting the EPS-producing capabilities of *W. confusa* stains [14,24], underscoring their potential for diverse applications. This reinforces the significance of EPS production in W1 and W2, as it can enhance their efficacy by improving gut colonization and persistence, thereby making them suitable for probiotic applications and other functional roles within microbial ecosystems.

#### 2.2.4. Safety Assessment

(a) Hemolysis Test

**Figure 4:**
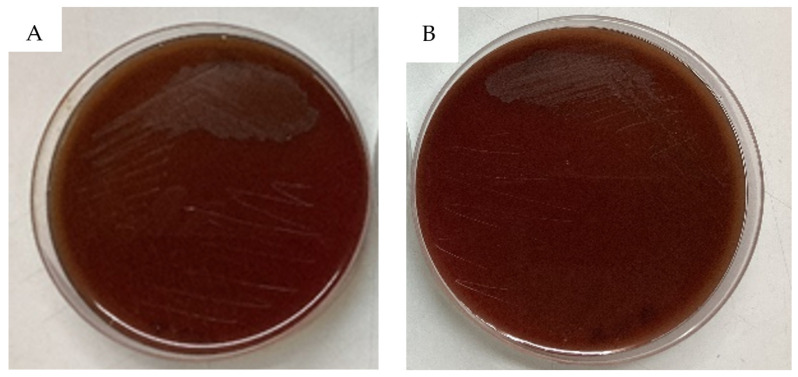
Hemolysis test on 5% defibrinated blood agar (**A**) W1 and (**B**) W2.

There is no hemolysis zone (gamma hemolysis) around the colonies on blood agar-enriched medium with sterile 5% defibrinated blood after 48 h of incubation at 37 °C (Figure 4). The absence of hemolytic activity is considered a positive trait for probiotic strains, as it indicates a reduced potential for causing harm to the host [24], suggesting that W1 and W2 strains are safe for consumption, which is a critical consideration in selecting probiotic strains.

(b) Antibiotic Susceptibility Test

*W. confusa* strains W1 and W2 were assessed for their antibiotic susceptibility using the agar disc-diffusion method. Both strains were susceptible to five out of seven antibiotics: ampicillin, erythromycin, chloramphenicol, tetracycline, and clindamycin. However, they were resistant to gentamycin and streptomycin (Table 1), which is similar to what other research has found with other *W. confusa* strains and *Weissella* species [11,13,25]. These findings suggest that *W. confusa* strains possess intrinsic resistance to certain antibiotics, potentially contributing to their ecological niche within the microbiota. Despite resistance to specific antibiotics, the overall susceptibility profiles support the safety and suitability of these strains for probiotic applications. Such natural resistance mechanisms may confer advantages in maintaining probiotic populations amid antibiotic exposure, mitigating safety concerns.

#### 2.2.5. Production of Antimicrobial Substances

**Figure 5:**
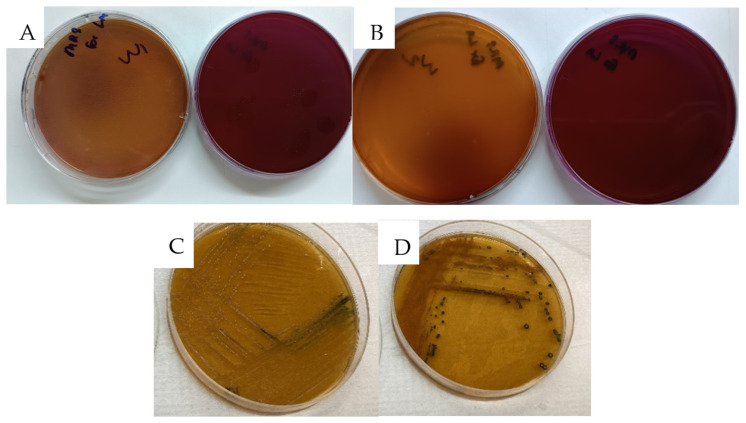
Production of antimicrobial substances (**A**) acid production of W1, (**B**) acid production of W2 (left: our studied culture, right: control agar plate), (**C**) H_2_O_2_ production of W1, and (**D**) H_2_O_2_ production of W2.

Yellow halos surrounding colonies, indicative of acid production, were observed on MRS bromocresol plates after incubation at 37 °C for 24 h (Figure 5A,B). This ability is a common trait of lactic acid bacteria, renowned for their fermentation capabilities that lead to the production of acidic byproducts [6]. The production of acid, including lactic acid, lowers the environmental pH, thereby effectively inhibiting the growth of pathogenic bacteria [26]. This characteristic is particularly important as it enhances the probiotic potential of W1 and W2 by creating an unfavorable environment for pathogens, promoting a balanced microbiota [27].

In addition, the colonies grown on modified MRS agar supplemented with 0.01 mg/mL horseradish peroxidase (HRP) and 0.25 mg/mL tetramethylbenzidine (TMB) showed a visible blue color after exposure to air for more than 3 h, which is indicative of hydrogen peroxide (H_2_O_2_) production (Figure 5C,D). H_2_O_2_ is known for its antimicrobial activities and is often associated with its ability to inhibit the growth of pathogenic bacteria, as demonstrated in previous research [25,28].

These findings align with prior studies that highlight the antimicrobial activities of lactic acid bacteria through the production of organic acids and H_2_O_2_ [28]. The dual action of acid and H_2_O_2_ production by W1 and W2 indicates a robust mechanism for controlling pathogen populations in the gut. This dual-production mechanism is vital as it can potentially improve the therapeutic applications of these strains in preventing and managing infections within the gastrointestinal tract. By contributing to a hostile environment for pathogens, W1 and W2 can help maintain the health of the gut microbiota and enhance overall gastrointestinal health.

#### 2.2.6. Antibacterial Activity

As shown in Table 2, the cell-free supernatants (CFS) of W1 and W2 exhibited significant antibacterial activity against *Stenotrophomonas maltophilia* DMST 19079 (*S. maltophilia*), *Salmonella enterica* serotype Typhi DMST 22842 (*Sa. enterica* serotype Typhi), *Vibrio cholerae* DMST 2873, and Carbapenem-resistant *Acinetobacter baumanii* (CRAB) SK005. Notably, the largest diameter of the inhibition zone was observed against *S. maltophilia* DMST 19079 (13 ± 1.7 mm and 12.3 ± 2.3 mm for W1 and W2, respectively). Conversely, the smallest inhibition was observed for CRAB SK005. However, it is noteworthy that the antimicrobial activity against CRAB SK005 was comparatively lower, suggesting potential differences in susceptibility among the tested pathogens.

Acid and H_2_O_2_ were the main antimicrobial substances produced by strains W1 and W2, demonstrating notable efficacy against these Gram-negative pathogenic bacteria. This finding aligns with prior research [25,26,29] highlighting the antimicrobial capabilities of probiotic bacteria stemming from acid and H_2_O_2_ production. The figures of the agar-well diffusion test can be seen in Supplementary Figure S2.

Overall, these findings support the potential antimicrobial capabilities of strains W1 and W2, which are attributed to their ability to produce acidic byproducts and H_2_O_2_ as part of their defense mechanism against pathogens. The observed production of antimicrobial substances further supports these strains’ probiotic potential by contributing to their protection against harmful bacteria. Additionally, their ability to inhibit a broad range of pathogens may support their use in preventing infections in various parts of the body, thus contributing to overall health and well-being [27,28,30].

### 2.3. Genomic Analysis

#### 2.3.1. Genome Features

The genome sizes of W1 and W2 were 2,380,646 and 2,203,909 bps with G + C contents of 44.62% and 44.78%, respectively. To ascertain the taxonomic classification of the species, the average nucleotide identity (ANI) analysis was conducted against 22 *Weissella* species sourced from the National Center for Biotechnology Information (NCBI) database. The ANI values of W1 and W2 strains against *W. confusa* VTT E-133279 (GCA_004771075.1, reference strain from the NCBI database) were 97.73% and 97.95%, respectively. The detailed features of the assembled genome are illustrated in Table 3. The genome completeness of our strains was over 98%, as shown in Supplementary Figure S1. These findings underscore the high quality and completeness of the genomic data obtained for strains W1 and W2, facilitating accurate taxonomic classification and comprehensive genomic analysis.

#### 2.3.2. Genomic Safety Assessment

No antimicrobial resistance (AMR) genes were detected in either the ResFinder database (80% identity threshold and 60% minimum length) or CARD’s RGI database (80% identity threshold with the parameter-perfect, strict, and loose hits). Moreover, the VFDB database showed five genes as predicted virulence genes for both W1 and W2 strains (Table S1). However, these genes are associated with adherence, antiphagocytic properties, and stress survival, which could potentially contribute to a probiotic’s ability to survive in the gut [20]. It is worth noting that these genes have been previously identified in published probiotics and non-pathogenic bacteria, as they are more related to defensive responses than offensive activities [20,21,31,32]. Therefore, we predicted that there would be no virulence gene that was harmful to the hosts. Additionally, strains W1 and W2 did not possess any virulence factors or genes associated with the pathogenesis in the analysis performed with Islandviewer 4. The lack of transferable antibiotic-resistance genes and virulence genes were a positive indication of the safety and suitability of the probiotic’s application.

#### 2.3.3. Prophage and CRISPR-Cas

**Figure 6:**
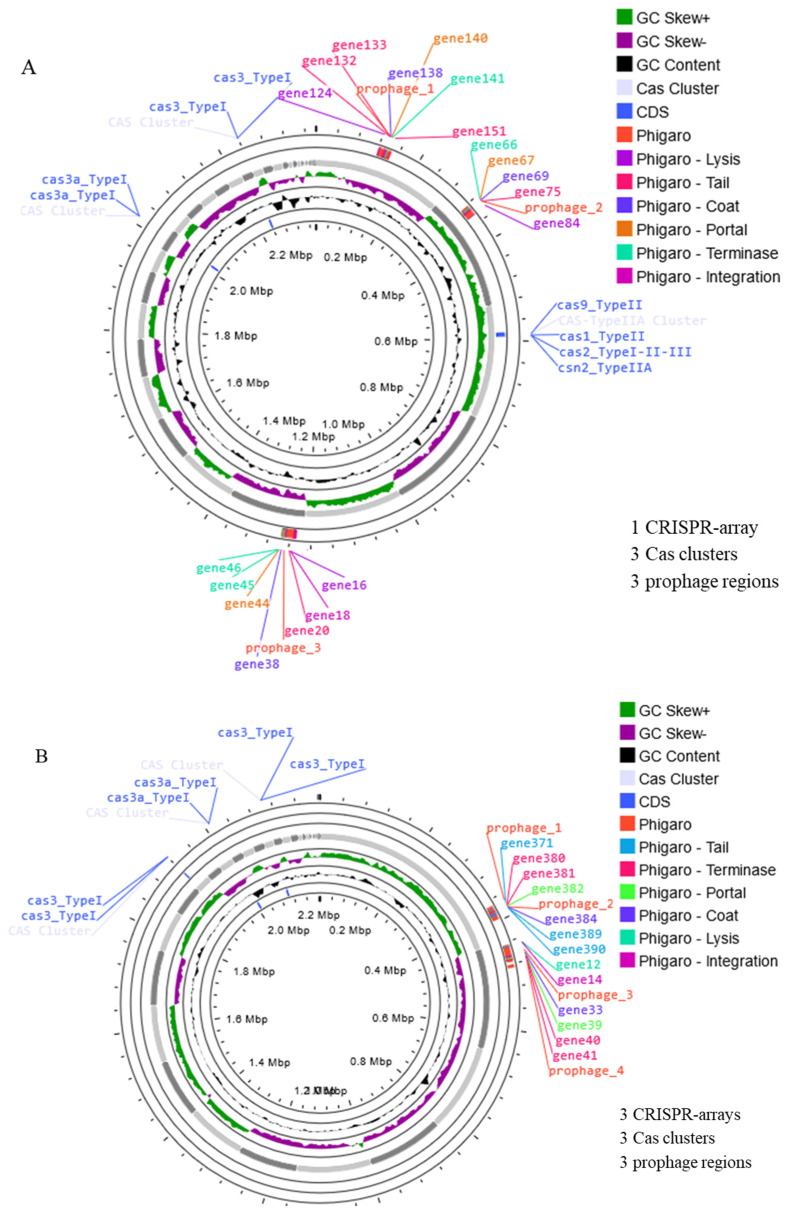
Circular Genome Maps of (**A**) *W. confusa* W1 and (**B**) *W. confusa* W2, illustrating CRISPR–Cas cluster and prophage. The figures were generated by CGview tool.

Prophages are viruses that infect bacteria and are integrated into the bacterial genome. They may contribute to immunity against related phages and bacterial evolution. However, prophages can also carry genes that encode virulence factors or antimicrobial resistance, which could be a concern for probiotic applications [33]. Screening for prophage sequences using Phigaro revealed the presence of three intact prophage regions in strain W1 and four intact prophages in W2, each containing the DNA packaging/head/tail gene cluster and the lysis cassette (Figure 6). However, no genes associated with AMR or virulence were detected within these prophage regions. It is notable that phages are also commonly found in many bacteria, including the *Lactobacillus* species and *Weissella* species [33,34]. Although the presence of prophage regions in the genome of bacteria could have both positive and negative implications for their potential use as probiotics, depending on the presence or absence of virulence factors or AMR [33], the absence of such genes within the prophage regions of strains W1 and W2 is a positive finding. It suggests that the prophages in strains W1 and W2 are unlikely to contribute to the virulence or antibiotic resistance of these bacteria, which is crucial for their safety as potential probiotics.

Furthermore, another adaptive immune system, the Clustered Regularly Interspaced Short Palindromic Repeats (CRISPR-Cas (CRISPR associated protein)), was analyzed using CRISPR-CAS++. The CRISPR–Cas system is known as adaptive immunity against foreign genetic elements, suggesting a role in defending against invading foreign genetic elements [35]. In *W. confusa* strain W1, one CRISPR array was associated with Cas3_typeI, one region was associated with Cas3a_TypeI, and another region was associated with Cas9_TypeII, while Cas1_TypeII and Csn2_TypeIIA were also identified. The association of different Cas proteins with specific CRISPR arrays indicates a potentially robust defense mechanism. On the other hand, *W. confusa* strain W2 appears to rely mainly on the Cas3a_TypeI-associated CRISPR regions for defense. Approximately 40% of bacteria and 90% of archaea have CRISPR–Cas systems, which are adaptive immune systems [20]. The presence of these CRISPR–Cas systems in *W. confusa* strains W1 and W2 highlights their ability to adapt and defend against invading genetic elements, which could be advantageous for their immunity and survival [35].

#### 2.3.4. Functional Prediction

**Figure 7:**
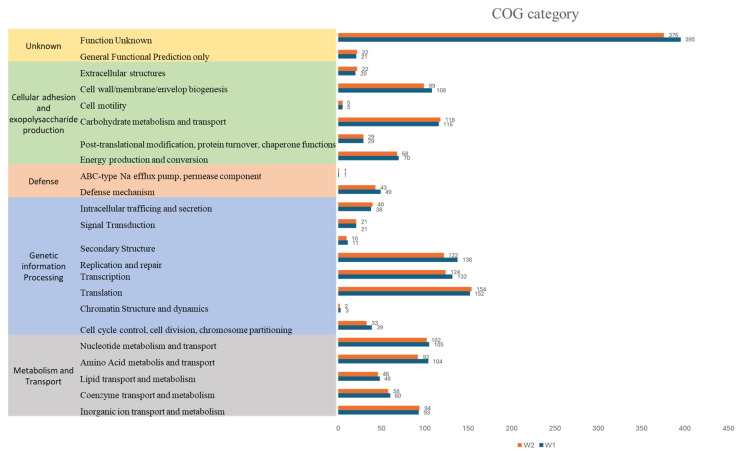
Functional analysis of gene families of *W. confusa* W1 and W2 based on distribution of Cluster of Orthologous group (COG) categories.

Clusters of Orthologous Groups (COGs) analysis was performed using the eggnog mapper web tool, revealing 23 clusters, as shown in Figure 7. The predominant category for both strains was “function unknown”, with 395 genes assigned to strain W1 and 376 genes to strain W2, underscoring the distinctive genomic features of these strains. Moreover, prominent functional groups implicated in crucial biological processes were revealed. Notably, functional groups implicated in cell adherence and EPS production were prominent, including genes related to extracellular structures (20 genes in W1 and 22 genes in W2), cell motility (five genes in both strains), and cell wall/membrane/envelope synthesis (108 genes in W1 and 99 genes in W2), carbohydrate metabolism and transport genes (116 genes in W1 and 118 genes in W2), energy production and conversion genes (70 genes in W1 and 68 genes in W2), and genes involved in post-translational modification and protein turnover (29 genes in both strains) [21,36]. These functional categories collectively accounted for a significant portion, approximately 19% and 20% of the total gene content for both W1 and W2 strains.

Additionally, 49 genes in W1 and 43 genes in W2 were identified as being associated with defense mechanisms, potentially providing adaptive advantages across various ecological niches. Other genes were related to genetic information processing, metabolism, and transport. These findings collectively highlight comprehensive insights into the genomic landscape of these strains, shedding light on their functional repertoire and potential ecological significance.

#### 2.3.5. Genes Related to Probiotic Properties

A significant number of gene-encoding proteins that are involved in stress responses, including those related to heat, cold, pH, bile tolerance, and adherence, were identified in strains W1 and W2. Specifically, there were nine genes associated with stress responses, four genes for heat shock, three genes for cold shock, two genes for bile tolerance, 11 genes for acid-stress responses, and 12 genes for adherence. Further details can be found in Table 4, where genes were identified based on the annotation results from both Prokka and eggNOG-mapper.

The genetic characteristics observed in *W. confusa* strains W1 and W2, which are related to the tolerance to gastrointestinal conditions, seem to support the high survival rates demonstrated in phenotypic assays (Figure 1). These characteristics are crucial for surviving in acidic conditions, such as those found in the stomach, and in the harsh bile environment of the gut [15,21].

Similarly, the high level of bacterial adhesions of W1 and W2 strains to the Caco2 cell line in the in vitro assay (Figure 2) aligns with the presence of various adherence-related genes. The number of genes related to adhesion ability was higher, including sortases, which play a crucial role in attaching bacteria to epithelial cells, fibronectin-binding proteins, and those responsible for EPS production. Previous studies on *Weissella* strains and other probiotic strains have also highlighted the effectiveness and significance of these adherence genes for the bacterial adhesion to epithelial cells [20,21,23,37]. It is important to note that while adherence is often considered a virulence factor in pathogenic bacteria, its presence alone may not fully explain virulence. Pathogenic strains typically require the presence of other factors, in addition to adherence ability, to effectively cause disease [38].

These integrated findings suggest that these W1 and W2 isolates are well-equipped to survive in the gut and other hostile environments and adhere to intestinal cells that are the main characteristics of probiotic bacteria.

#### 2.3.6. EPS Production-Related Gene Cluster

EPS production is a crucial attribute of probiotic bacteria, serving to protect the organism against environmental stresses and facilitate attachment to host intestinal cells [39]. Additionally, EPS produced by beneficial bacteria holds significant promise in various food industry applications, serving as viscosifying agents, stabilizers, and emulsifiers. These compounds also offer notable health benefits, including the inhibition of pathogenic organism colonization, and a prebiotic effect [14,30,40].

**Figure 8:**
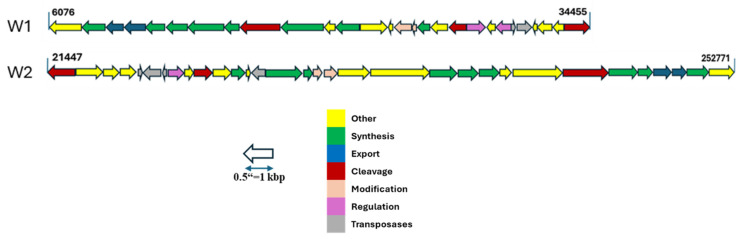
Illustration of exopolysaccharide-production clusters of *W. confusa* W1 and W2 strains. (Gene functions were predicted by Prokka and protein functions were defined by UniProt.).

To gain insight into the characteristics of the EPS production gene cluster, a detailed analysis of an EPS biosynthesis cluster adjacent to the *epsL* gene in both strains was conducted. This cluster spans 28,444 bps and 38,226 bps in node 18 of W1 and node 1 of W2 strains, respectively (Figure 8), encompassing regions associated with regulation, synthesis, modification, and export systems that are essential components of a gene cluster, as demonstrated in Nourikyan et al. [41]. Detailed gene information can be seen in Supplementary Table S2.

Furthermore, the observed EPS gene cluster provides a better understanding of the robust exopolysaccharide production observed in our in vitro analysis (Figure 3). With the growing interest in the industrial applications of EPS produced by *Weissella* strains, the availability of detailed analysis data regarding the EPS cluster remains limited [23,40]. Therefore, our findings regarding the EPS cluster in *W. confusa* strains represent a significant contribution to the field. This analysis not only enhances our understanding of the genetic basis of EPS production in these strains but also suggests potential applications in the food industry and inhibition of pathogenic organism colonization [30].

#### 2.3.7. Secondary Metabolic Products

**Figure 9:**
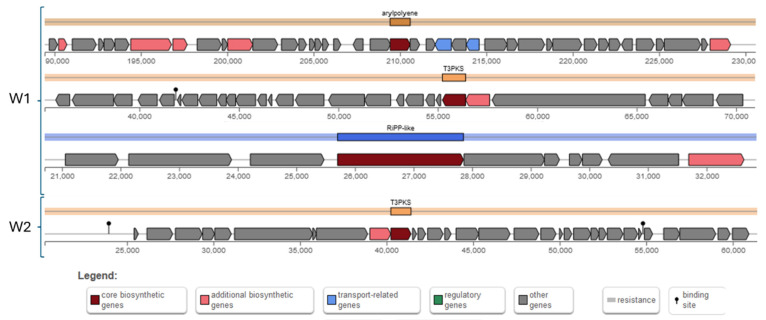
Illustrating the gene clusters encoding the secondary metabolic enzymes in the genomes of *W. confusa* W1 and W2.

Secondary metabolites have gained interest not only due to bacterial adaptation and protection, but also due to their beneficial effects for the hosts, such as antibacterial and anticancer activities [42,43]. Therefore, in this study, the production of a limited number of secondary metabolites (Figure 9) was explored by using the antiSMASH tool. The W1 strain possesses an aryl polyene gene cluster, a T3PKS (Polyketide synthase) cluster, and an unspecified RiPP (ribosomal synthesized and post-translationally modified peptide) cluster. The aryl polyene gene cluster encodes 40 genes spanning 41,167 nucleotides and is associated with the production of aryl polyenes, which are known for protecting bacteria from reactive oxygen species [44]. The T3PKS cluster consists of 28 genes spanning 35,717 nucleotides and is involved in the biosynthesis of polyketides, which influence a wide range of biological activities, including antimicrobial, anticancer, and immunosuppressive effects [45]. The RiPP cluster comprises 10 genes spanning 12,148 nucleotides, and while the specific product is unspecified, RiPPs are a diverse class of natural products that often exhibit antimicrobial activities and contribute to competition within microbial communities for niches [46].

In contrast, the W2 strain possesses only a T3PKS cluster, which encodes 31 genes spanning 41,167 nucleotides. This cluster is also involved in the biosynthesis of polyketides, suggesting that the W2 strain may produce polyketide compounds with similar biological activities as those produced by the W1 strain. However, Bagel4 confirmed that neither strain has a bacteriocin-production gene.

The identification of these gene clusters implies the capacity for generating a range of secondary metabolites, which may confer advantageous traits such as stress tolerance and antibacterial efficacy on these strains. The discovery of aryl polyene and T3PKS gene clusters aligns with what Yuan et al. [33] observed in other *W. confusa* strains and is prevalent among beneficial bacteria, including *Lactobacillus plantarum* [20,34].

#### 2.3.8. Data Summarization

**Figure 10:**
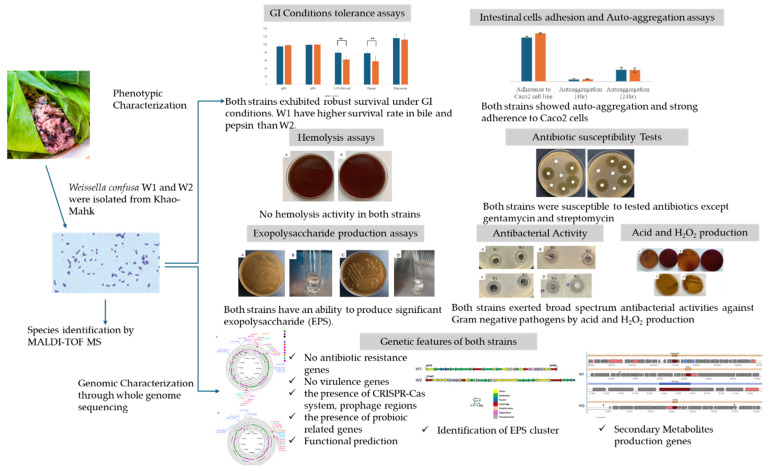
Summary of isolation, phenotypic and genetic characteristics of *Weissella confusa* strains W1 and W2 from Khao-Mahk in Thailand.

This study demonstrated that *W. confusa* strains W1 and W2 possess key probiotic characteristics, including a tolerance to gastric and intestinal conditions, adhesion to intestinal cells, and safety for hosts, which is supported by both phenotypic assays and whole genome analysis. These strains also exhibited robust EPS production and significant antibacterial activity through acid and H_2_O_2_ production. Figure 10 illustrates the summary of our findings, showcasing the phenotypic results and genetic characteristics of *W. confusa* strains W1 and W2.

## 3. Material and Methods

### 3.1. Bacteria Isolation and Identification

Bacterial isolation and identification were conducted in accordance with Chen et al. [47]. Bacterial strains were collected from 1 g of Khao-Mahk-fermented glutinous rice in 15 mL tubes, which contained 5 mL of De Man, Rogosa, Sharpe (MRS) broth (HiMedia, Nashik, India) and cultured on MRS agar (HiMedia, Nashik, India) in anaerobic jars at 37 °C for 2 days. Species identification was done by MALDI-Biotyper (Bruker Daltonics GmbH, Bremen, Germany) and kept in broth with 20% glycerol at −80 °C. The cultures, which were cultivated in MRS broth and incubated at 24 h at 37 °C, were centrifuged at 10,000× *g* for 5 min and filtered the supernatant through 0.2 µm membrane filter. After that, the filtrate was used as non-neutralized cell-free culture supernatants (CFS), which are being kept at −20 °C for further analysis.

For antibacterial activity testing, *S. maltophilia* DMST 19079, *Sa. enterica* serotype Typhi DMST 22842 and *V. cholerae* DMST 2873 were purchased from the Department of Medical Science, Thailand (DMST). CRAB SK005 was clinical strain from Southern Thailand [48]. Reference strains from the American Type Culture Collection (ATCC) were also used, including *Escherichia coli* ATCC 25922, *Staphylococcus aureus* ATCC 25923, and *Staphylococcus aureus* ATCC 29213. These pathogen stains were cultured on Tryptone Soya agar (TSA) (HiMedia, Nashik, India).

### 3.2. Determination of Probiotic Properties

#### 3.2.1. Determination of Bile Tolerance

Determination of bile tolerance was done according to Rodríguez et al. [49], with slight modification. Potential probiotic cultures in MRS broth were cultured for 4 h and prepared to 0.5 ± 0.02 McFarland to standardize the cells with 3 mL of MRS broth. The cell suspensions were prepared in MRS broth containing 0.3% (*w*/*v*) bile salt (Sigma–Aldrich, St. Louis, MO, USA). The number of colony-forming units (CFU/mL) present were determined first and incubated at 37 °C for 4 h. The suspensions were plated onto MRS agar to determine colony-forming units (CFU/mL) present after exposure to varying bile concentrations. Survival rates were determined as

Survival rate = (Final (log CFU/mL)/Initial (log CFU/mL)) × 100

#### 3.2.2. Determination of Acid Tolerance

The acid-resistant strains were prepared in the same way as the acid-tolerance assay according to Mulaw et al. [50]. The cell suspensions were adjusted to varying acidity of pH 3 and pH 4, with HCl and NaOH in 3 mL of MRS broth. The cultures in the test tubes were incubated at 37 °C for up to 3 h. The strains were plated to determine the colony-forming units (CFU/mL) present after exposure.

Survival rate = (Final (log CFU/mL)/Initial (log CFU/mL)) × 100

#### 3.2.3. Detection of Pepsin and Pancreatin Tolerance

According to Romyasamit et al. [51], with slight modification, 2 g/L of pepsin solution were prepared by adding pepsin (Sigma–Aldrich, St. Louis, MO, USA) in MRS broth, and the pH was adjusted to 3.0 with HCL. Then, 1 g/L pancreatin solution was prepared by suspending pancreatin (Sigma–Aldrich, St. Louis, MO, USA) in MRS broth at pH 8.0. Potential probiotic cultures in MRS broth were cultured for 4 h and prepared to 0.5 ± 0.02 McFarland at OD_600_ to standardize the cells with 3 mL of MRS broth with pepsin/pancreatin, respectively, and inoculated into MRS broths containing already prepared pepsin or pancreatin and incubated at 37 °C for 3 or 4 h. The strains were plated to determine the number of colony-forming units (CFU/mL) present before and after exposure to enzymes.

Survival rate = (Final (log CFU/mL)/Initial (log CFU/mL)) × 100

#### 3.2.4. Auto-Aggregation

Test for auto-aggregation was done according to Somashekaraiah et al. [52]. The W1 and W2 strains, cultured overnight in MRS broth, were rinsed twice and resuspended in PBS solution (pH 7.4) (HiMedia, Nashik, India) at an absorbance of 0.5 ± 0.02 at OD_600_. The cell suspensions were mixed thoroughly and incubated aerobically at 37 °C. UV VIS Spectrophotometer (Shimadzu, Kyoto, Japan) was used to measure the absorbance of the upper suspension for 0,2,4, and 24 h. Each isolate’s auto-aggregation ability expresses, in percentage (%), the formula 100 × [1 − OD1/OD2], where OD1 represents the absorbance of the strains at 4 or 24 h and OD2 represents the absorbance of the potential probiotic strains prior to incubation.

#### 3.2.5. Exopolysaccharide Production Activity

Exopolysaccharide production activity was detected by incubating W1 and W2 isolates on MRS agar supplemented with 2% (*w*/*v*) sucrose (Amresco, OH, USA), as Ruas–Madiedo et al. and Paulo et al. [22,53] did. After 48 h of incubation at 37 °C anaerobically, the production of exopolysaccharides was indicated by the development of a translucent or creamy material involving a mucoid colony on agar medium or long filaments. The production of polymers was confirmed by adding each colony in absolute alcohol. The EPS was indicated by precipitate formation [22].

#### 3.2.6. Determination for Adherence to Intestinal Epithelial Cells

Adhesion of W1 and W2 strains was measured according to Monteagudo–Mera et al. [54]. W1 and W2 isolates were washed twice and resuspended in fresh DMEM (Thermo Fisher Scientific, Waltham, MA, USA) to 10^7^ CFU/mL. These suspensions were applied to each well of a tissue culture plate that contained a Caco-2 monolayer in equal quantities (1 mL). After that, the plate was incubated in an environment with 5% CO_2_ for 2 h at 37 °C. Following incubation, the monolayers were washed four times with sterile PBS to get rid of any unattached bacteria. Subsequently, the adherent bacteria were extracted using 800 μL of sterile, ultra-pure water and pipetted repeatedly after adding 200 μL of 2.5% w/v trypsin (Sigma–Aldrich, St. Louis, MO, USA) to separate the monolayer. Enumeration of adhered bacteria was performed by plating serial dilutions on TSA. Adhesion values (%) were calculated using the formula (V0 − V1)/V0 × 100, where V0 is the initial viable count of tested bacteria and V1 is the viable bacteria count obtained from the Caco-2 cells at the end of the experiment.

#### 3.2.7. Acid and H_2_O_2_ Production

As in Chen et al. [47], the ability to produce acid was evidenced by the presence of yellow halos surrounding colonies on MRS bromocresol plates (0.12 g/L bromocresol purple (Sigma–Aldrich, St. Louis, MO, USA) + 2% sucrose (Amresco, OH, USA) + 0.2 g/L sodium azide (Sigma–Aldrich, St. Louis, MO, USA)) after incubation 24 h anaerobically at 37 °C.

Similar to Song et al. [55], bacteria were cultured on modified MRS agar with 0.01 mg/mL horseradish peroxidase (HRP) (Sigma–Aldrich, St. Louis, MO, USA) and 0.25 mg/mL tetramethylbenzidine (TMB) (Sigma–Aldrich, St. Louis, MO, USA) at 37 °C for 48 h. After incubation, H_2_O_2_ producer strains were exposed to air in a safety cabinet. H_2_O_2_ production will be ranked as positive or negative according to the appearance of a visible blue color.

### 3.3. Phenotypic Safety Assessment

#### 3.3.1. Hemolytic Potential

Determination of hemolytic potential was done according to Foulquié Moreno et al. [56]. Briefly, the studied strains were streaked on blood agar-enriched medium (HiMedia, Nashik, India) with sterile 5% defibrinated blood and incubated at 37 °C for 48 h. The presence or absence of hemolysis zones was observed. *Staphylococcus aureus* ATCC 29213 was used as control to validate the test.

#### 3.3.2. Susceptibility to Antibiotics

Antibiotic susceptibility was evaluated by disc diffusion according to the standard criteria of CLSI 2020 against antibiotics recommended by the European Food Safety Authority. Then, 1 × 10^8^ CFU/mL of each strain in MRS broth were spread on MRS agar [51]. Antibiotic discs—ampicillin (AM; 10 µg), chloramphenicol (C; 30 µg), erythromycin (E; 15 µg), gentamycin (GM; 10 µg), streptomycin (S; 10 µg), tetracycline (TE; 30 µg), and clindamycin (HiMedia, Nashik, India) (CA; 2 µg), were placed on the medium and incubated at 37 °C for 24 h. Depending on the size of the zone of inhibition, the result was interpreted as resistance (R) for <14 mm, intermediate resistance (I) (15 < I < 20 mm), or susceptibility to antibacterial agents (S) (>20 mm). *E. coli* ATCC 25922 and *Staphylococcus aureus* ATCC 25923 were used for validation.

### 3.4. Determination of Antimicrobial Activities of Probiotic Isolates (Agar-Well Diffusion Method)

Antimicrobial activity of W1 and W2 strains was determined according to Nigam et al. [57], with slight modification. Overnight cultures of *S. maltophilia* DMST 19079, *Sa. enterica* serotype Typhi DMST 22842, *V. cholerae* DMST 2873, and CRAB SK005 were adjusted to a cell density of 0.5 McFarland in TSB and spread on Muller–Hinton agar. Wells with 7 mm in diameter were made in the agar. Then, 100 μL of cell-free supernatant of each isolated strain were added to each well. The agar plates were incubated at 37 °C for 24 h. The inhibition zones were measured using calipers.

### 3.5. Genomic DNA Extraction and Bioinformatics Analysis

Genomic DNA of *S. maltophilia* was extracted using Zymo DNA fungal/bacterial Microprep Kit (Zymo Research, Irvine, CA, USA) according to the manufacturer’s instructions. The concentration (>50 ng/µL) and purity (A260/280: 1.8–2.0) of the DNA were measured by using NanoDrop™ 2000 Spectrophotometer (Thermo Fisher Scientific, Waltham, MA, USA), and DNA quality was checked by 1.5% agarose gel electrophoresis.

Whole genome sequencing was done by short-read sequencing (150 bps paired-end reads) by MGISEQ 2000, which is a next-generation sequencing by the Beijing Genomics Institute in Shenzhen, China. Sequence reads were de novo assembled using SPAdes 3.15.5 [58]. The assembled genome quality and completeness were checked by Quast [59] and BUSCO v5.4.3 [60]. The assembled genome was subjected to Prokka and functional annotations on Rapid Annotation using the Subsystem Technology (RAST) server [61,62]. Clusters of Orthologous Groups (COGs) for pangenome of the studied isolates were obtained using the eggNOG mapper v2 web tool [36]. Protein functions were defined by UniProt [63]. These gene and protein functions were applied to generate probiotic properties related to genes in Table 4 and EPS cluster (Figure 8). Gene orientations were defined by using Geneious prime 2023.1.2, and the figures were generated by R program v4.3.2 [64,65]. Average nucleotide identity (ANI) value was calculated against the downloaded genomic sequences of each *Weissella* species from NCBI database by using fastANI v1.34 [66].

Antimicrobial resistance genes were predicted using the ResFinder 4.5.0 and CARD’s RGI 6.0.3 by using 80% identity as cut-off values [67,68]. Then, virulence-associated genes were detected by using cut-off values of 80% identity and 1e-30 E-value cut-offs against the virulence factor database (VFDB) [69]. IslandViewer v4 was used to predict the pathogenicity of the bacteria toward human hosts [70]. The secondary metabolite biosynthesis gene clusters were annotated and analyzed using the Antibiotics and Secondary Metabolite Analysis Shell (antiSMASH V6.0) web server with “relaxed” detection strictness, and figures were also downloaded [71]. Bacteriocin-producing gene was detected by bagel4 [72]. Phage regions were produced using Phigaro2.3.0 [73]. CRISPR–CAS system was revealed by using CRISPR–CAS ++1.1.2 [74]. Circularized genome visualization was done by using CGview tool v1.0 [75].

### 3.6. Statiscal Analysis

All statistical analyses were conducted using a two-proportion Z-test by R program v4.3.2 [65]. The significance level was set at *p* < 0.05 to determine the statistical significance of the differences observed between the strains.

## 4. Conclusions

This study explored *W. confusa* strains W1 and W2 as promising probiotic candidates due to their phenotypic and genomic traits, as well as safety. Notably, these strains demonstrated robust EPS production, which enhances their adherence and resistance to environmental stress, making them suitable candidates for applications in the food industry. Additionally, their antibacterial activity against various pathogens suggests their potential role in combating bacterial infections. While our findings lay the foundation for further exploration, additional research, such as in vivo studies, is warranted to assess the efficacy and safety of these strains in animal models. Nonetheless, *W. confusa* strains W1 and W2 present promising avenues for probiotic development and food safety enhancement.

## Figures and Tables

**Table 1:** Antibiotic susceptibility patterns of *W. confusa* strains W1 and W2.

Antibiotics	W1	W2
	Inhibition Zone (mm)	Inhibition Zone (mm)
Ampicillin (10 µg)	21.1 ± 1.5 (S)	20.8 ± 1.2 (S)
Erythromycin (15 µg)	23.3 ± 1.8 (S)	23.3 ± 1.8 (S)
Chloramphenicol (30 µg)	24.8 ± 2.0 (S)	24.5 ± 1.6 (S)
Gentamycin (10 µg)	9.8 ± 0.6 (R)	9.3 ± 0.8 (R)
Tetracycline (30 µg)	23.2 ± 2.0 (S)	22 ± 1.3 (S)
Streptomycin (10 µg)	0 (R)	0 (R)
Clindamycin (2 µg)	25.8 ± 2.1 (S)	24.8 ± 2.0 (S)

Tests were done on three independent experiments. S: Sensitive, R: Resistance.

**Table 2:** Determination of antibacterial activity of CFS of strains W1 and W2 against pathogens.

	Zone of Inhibition (mm) ± SD			
Strains	*S. maltophilia* DMST 19079	*Sa. enterica* Serotype Typhi DMST 22842	*V. cholerae* DMST 2873	CRAB SK005
W1	13 ± 1.7 mm	11.3 ± 1.5 mm	10.7 ± 2.3 mm	8.6 ± 1.2 mm
W2	12.3 ± 2.3 mm	11.0 ± 1.7 mm	10.0 ± 1.7 mm	9.7 ± 1.5 mm

Tests were done on three independent experiments.

**Table 3:** Genome features of *W. confusa* W1 and W2 strains.

	W1	W2
Total genome size (bp)	2,380,646	2,203,909
Total contigs (≥200 bp)	51	42
G + C (%)	44.62	44.78
Largest contig (bp)	277,000	438,208
N50	209,762	157,099
N90	37,460	31,601
L50	5	5
L90	17	14
CDS	2265	2131
rRNA	5	4
tRNA	81	74
ANI value with *W. confusa* VTT E-133279 from NCBI (%)	97.73	97.95

**Table 4:** List of genes related to putative probiotic properties identified in the *W. confusa* strains W1 and W2.

Genes	Function/Description	W1 (No. of Gene)	W2 (No. of Gene)
**Stress Response**			
*lepA2*	Accurate and efficient protein synthesis under certain stress conditions	1	2
*lepA*	Accurate and efficient protein synthesis under certain stress conditions	1	1
*uspA*	Universal stress protein UspA and related nucleotide-binding proteins	4	4
*uspA3*	Universal stress protein UspA and related nucleotide-binding proteins	1	1
*ctsR*	Firmicute transcriptional repressor of Class III stress genes	1	1
*typA*	Membrane GTPase involved in stress response	1	1
*obg*	An essential GTPase that plays a role in control of the cell cycle, stress response, ribosome biogenesis, differentiation, and morphogenesis	1	1
*hslO*	Redox regulated molecular chaperone for oxidative stress	1	1
*groL*	Prevents misfolding and promotes the refolding and proper assembly of unfolded polypeptides generated under stress conditions	1	1
**Heat Shock**			
*dnaK*	Heat-shock 70 kDa protein	1	1
*grpE*	Participates actively in the response to hyperosmotic and heat shock by preventing the aggregation of stress-denatured proteins, in association with DnaK and GrpE	1	1
*hrcA*	Negative regulator of Class I heat-shock genes (grpE-dnaK-dnaJ and groELS operons). Prevents heat-shock induction of these operons	1	1
*yabO*	Ribosome-associated heat-shock protein implicated in the recycling of the 50S subunit	1	1
**Cold Shock**			
*cspB*	Cold-shock protein	1	1
*cshB*	Conjunction with the cold-shock proteins to ensure proper initiation of transcription at low and optimal temperatures	1	1
*cspC*	Cold-shock proteins	1	1
**Bile Tolerance**			
*mleP*	Sodium Bile acid symporter family	1	1
*recU*	Endonuclease for DNA repair, homologous recombination, and chromosome segregation	1	1
**Acid Stress**			
*nhaC*	Sodium/hydrogen antiporter	1	1
*nhaP3*	NhaP-type Sodium/hydrogen and Potassium/hydrogen antiporters	0	2
*atpC*	Produces ATP from ADP in the presence of a proton gradient across the membrane	1	1
*atpD*	Produces ATP from ADP in the presence of a proton gradient across the membrane	1	1
*atpG*	Produces ATP from ADP in the presence of a proton gradient across the membrane	1	1
*atpA*	Produces ATP from ADP in the presence of a proton gradient across the membrane	1	1
*atpH*	Produces ATP from ADP in the presence of a proton gradient across the membrane	1	1
*atpF*	Produces ATP from ADP in the presence of a proton gradient across the membrane	1	1
*atpE*	Produces ATP from ADP in the presence of a proton gradient across the membrane	1	1
*atpB*	Produces ATP from ADP in the presence of a proton gradient across the membrane	1	1
*nhaK22*	Sodium/hydrogen exchanger family	1	1
**Adherence**			
*gap*	Glyceraldehyde-3-phosphate dehydrogenase	1	1
*brpA*	Biofilm regulatory protein A	1	0
*cps2I*	Psort location Cytoplasmic Membrane, score	1	0
*srtC/srtA*	Sortase family	2	2
*fbpA*	fibronectin binding protein	1	1
*tuf*	elongation factor Tu	1	1
*pgaC1*, *pgaC2*	Poly-beta-1/2,6-N-acetyl-D-glucosamine synthase	2	2
*eno*	Enolase	1	1
*epsH*	Putative glycosyltransferase epsH	1	1
*epsL*	Putative sugar transferase	1	1

## Data Availability

The genomic sequences of *W. confusa* W1 and W2 have been submitted to the NCBI genome submission portal under Biosample SAMN41274410 and SAMN41274411 in Bioproject PRJNA1109523 on 9 May 2024.

## Supplementary Materials

The following supporting information can be downloaded at: https://www.mdpi.com/article/10.3390/antibiotics13070604/s1, Figure S1: Genome completeness of *W. confusa* W1 and W2 analyzed by BUSCO; Figure S2: Antibacterial activity of CFS of *W. confusa* W1 and W2 strains against (A) *Salmonella enterica* serotype Typhi DMST 22842 (B) *Vibrio cholerae* DMST 2873 (C) Carbapenem-resistant *Acinetobacter baumanii* SK005 (D) *Stenotrophomonas maltophilia* DMST 19079 using agar-well diffusion method; Table S1: Predicted virulence genes in VFDB database; Table S2: Gene information involved in exopolysaccharide gene clusters
