# Supplementary material for: Exploring Weissella confusa W1 and W2 Strains Isolated from Khao-Mahk as Probiotic Candidates: From Phenotypic Traits to Genomic Insights
Source: Antibiotics (Basel). 2024 Jun 28;13(7):604. doi: 10.3390/antibiotics13070604 (PMC11273482; doi:10.3390/antibiotics13070604)
Supplement: Supplementary file 1 [file antibiotics-13-00604-s001.zip › antibiotics-3072038-supplementary.pdf]

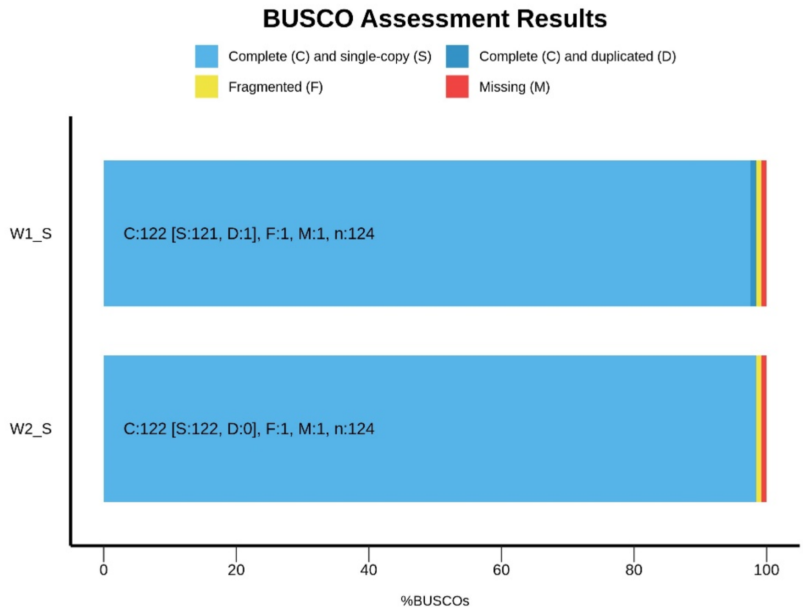

Figure S1. Genome completeness of *W. confusa* W1 and W2 analyzed by BUSCO

Table S1. Predicted virulence genes in VFDB database.

**Genes**	Function	W1 (No of genes)	W2 (No of genes)
**Antiphagocytic**			
gnd	6-phosphogluconate dehydrogenase, decarboxylating	1	1
galE	Polysaccharide capsule	1	1
Tig/ropA	Trigger factor	1	1
ndk	Nucleoside diphosphate kinase	1	1
**Adherence**			
gap	Glyceraldehyde-3-phosphate dehydrogenase	1	1

Table S2. Gene information involved in exopolysaccharide gene clusters

Strains	Gene name in sequences (prokka)	Gene name (uniprot)	Functions	Region	Functional category	Size (bp)
W1 (node 18)	Hypothetical protein	*RfbX*	Other Cellular Component	7,560 -> 6,076	other	1485
	Hypothetical protein	*FGL80_07090*	Glycosyltransferase	8,807 -> 7,680	synthesis	1128
	tagG	*tagG*	ABC-2 type transporter transmembrane domain-containing protein	9,648 -> 8,821	Export/import /regulation	828
	btuD 3	*btuD 3*	ABC type transporter	10,625 -> 9,654	Export/import /regulation	972
	Hypothetical protein	*H9L19_06025*	Glycosyltransferase family 2 protein	11,689 -> 10,802	synthesis	888
	putative glycosyltransferase	*IV68_GL000138*	Glycosyltransferase	12,731 -> 11,766	synthesis	966
	Hypothetical protein	*WOSG25_080230*	Glycosyltransferase RgtA/B/C/D-like domain-containing protein	14,536 -> 12,818	synthesis	1719
	Hypothetical protein	*E9934_11360*	Glycosyltransferase RgtA/B/C/D-like domain-containing protein	15,291 -> 14,590	synthesis	702
	Hypothetical protein	*lytG_2, QX99_01554*	Hydrolase activity, extracellular component	17,413 -> 15,476	cleavage	1938
	Hypothetical protein	*H9L19_07490*	Glycosyltransferase activity	19,499 -> 17,436	synthesis	2064
	Hypothetical protein	*WOSG25_080270*	Lipoprotein (other cellular component)	20,056 -> 19,541	other	516
	glfC	*glfC*	UDP-glucose 4-epimerase, exopolysaccharide (EPS) biosynthesis	21,362 -> 20,184	Synthesis	1179
	Hypothetical protein	*SAMN05216341_102152*	Predicted membrane protein (cellular component)	21,630 -> 22,985	other	1356
	Hypothetical protein	*FC34_GL001529*	GlsB/YeaQ/YmgE family stress response membrane protein (plasma membrane)	23,433 -> 23,185	other	249
	Hypothetical protein	*BN623_01340*	Acyltransferase 3 domain-containing protein	24,342 -> 23,509	modification	834
	Hypothetical protein	*PIROE2DRAFT_3857*	acyltransferase activity, (acyltransferases can be involved in the modification of EPS	24,548 -> 24,393	modification	156

			molecules by adding acyl groups, such as fatty acids, to the polysaccharide backbone)			
	epsL	*epsL*	Putative sugar transferase EpsL, chain length determination or regulation.	25,303 -> 24,674	synthesis	630
	Hypothetical protein	*QX99_01550*	Hypothetical protein	26,126 -> 25,356	other	771
	map 2	*map 2*	Methionine aminopeptidase	26,970 -> 26,188	cleavage	783
	brp A	*brp A*	Biofilm regulating protein	27,171 -> 28,064	regulation	894
	Hypothetical protein	*WOSG25_080290*	Teichoic acid glycosylation protein	28,541 -> 28,113	other	429
	Hypothetical protein	*DW717_02355*	PucR family transcriptional regulator, Sugar diacid utilization regulator	29,317 -> 28,598	Transcription/ regulation	720
	Hypothetical protein	*FPFC 1*	Transposases, DNA binding	29,621 -> 29,881	Transposases	261
	ISWci2	*ISWci2*	IS3 family transposase ISWci2	29,929 -> 30,636	Transposases	708
	Hypothetical protein	*HMPREF0877_1823*	Helix-turn-helix domain protein (DNA Binding)	31,141 -> 30,938	other	204
	Hypothetical protein	*QX99_01496*	Helix-turn-helix domain protein (DNA Binding)	32,538 -> 31,813	other	726
	Hypothetical protein	*QX99_01496*	Helix-turn-helix domain protein (DNA Binding)	33,074 -> 32,553	other	522
	pepS	*pepS*	Aminopeptidase, hydrolase activity	33,217 -> 34,455	cleavage	1239
W2 (node 1)	*pepS*	*pepS*	Aminopeptidase, hydrolase activity	215,709 -> 214,477	cleavage	1157
	Hypothetical protein	*QX99_01496*	Helix-turn-helix domain protein	215,852 -> 217,114	other	1263
	Hypothetical protein	*QX99_01498*	Helix-turn-helix domain protein	217,786 -> 218,559	other	774
	Hypothetical protein	*QX99_01498*	Helix-turn-helix domain protein	218,549 -> 219,277	other	729

Hypothetical protein	*C1I99_32025*	Transposase	219,371 -> 219,556	Transposases	186
ISWci2	*FPFC_120030*	IS3 family transposase ISWci2	220,546 -> 219,752	Transposases	795
Hypothetical protein	*FPFC_120020*	Transposase	220,854 -> 220,594	Transposases	261
Hypothetical protein	*DW717_02355*	PucR family transcriptional regulator	221,158 -> 221,877	Transcription/ regulation	720
Hypothetical protein	*WOSG25_080290*	Teichoic acid glycosylation protein	221,934 -> 222,362	other	429
map 1	*map 1*	Methionine aminopeptidase	222,586 -> 223,368	cleavage	783
Hypothetical protein	*FY536_01520*	DUF4422 domain-containing protein (unknown)	223,430 -> 224,200	other	771
epsL	*epsL*	Putative sugar transferase EpsL, chain length determination or regulation.	224,253 -> 224,882	synthesis	630
Hypothetical protein	*IRY55_04125*	GlsB/YeaQ/YmgE family stress response membrane protein	224,970 -> 225,218	other	249
Hypothetical protein	*LB941_12275*	ISL3 family transposase	226,206 -> 225,646	transposases	561
Hypothetical protein	*H9L19_07515*	CDP-glycerol glycerophosphotransferase family protein	227,100 -> 228,707	synthesis	1608
tarD	*C270_02975*	Glycerol-3-phosphate cytidylyltransferase	228,725 -> 229,135	synthesis	411
putative glycosyltransferase	*H0267_10780*	Glycosyltransferase family 2 protein	229,266 -> 230,204	synthesis	939
Hypothetical protein	*HMPREF0877_1596*	Arylsulfatase (hydrolase)	230,207 -> 230,623	Modification (remove sulfate groups of precursors)	417
Hypothetical protein	*HMPREF0877_1596*	Arylsulfatase, 3.1.6.-	230,696 -> 231,241	Modification (remove sulfate groups of precursors)	546
Hypothetical protein	*QX99_01574*	Phosphoglycerol transferase, alkaline phosphatase superfamily	231,213 -> 232,694	other	1482
Hypothetical protein	*SAMN04488506_0470*	Uncharacterized membrane protein YfhO	233,078 -> 235,822	other	2745

	glf	*glf*	Isomerase activity (UDP- galactopyranose mutase activity)	235,897 -> 237,075	synthesis	1179
	csbB	*H9L19_07650*	Glycosyltransferase family 2 protein	237,182 -> 238,114	synthesis	933
	Hypothetical protein	*WOSG25_080280*	Glycosyltransferase	238,212 -> 239,162	synthesis	951
	Hypothetical protein	*WOSG25_080270*	lipoprotein	239,307 -> 239,780	other	474
	Hypothetical protein	*WOSG25_080260*	Putative mannosyl-glycoprotein endo-beta-N-acetylglucosamidase	239,802 -> 241,985	other	2184
	Hypothetical protein	*B5F53_04560*	Peptidase M23 domain-containing protein	242,019 -> 244,085	Cleavage	2067
	Hypothetical protein	*FD46_GL000348*	Glycosyltransferase RgtA/B/C/D-like domain-containing protein	244,494 -> 246,380	synthesis	1887
	glfT1	*glfT1(H9L19_06025)*	Glycosyltransferase family 2 protein	246,396 -> 247,280	synthesis	885
	btuD1	*btuD1(H9L19_00885)*	ABC transporter ATP-binding protein	248,244 -> 249,215	Export/import /regulation	972
	tagG	*tagG(IV73_GL000915)*	ABC-2 type transporter transmembrane domain-containing protein	249,221 -> 250,048	Export/import /regulation	828
	Hypothetical protein	*FGL80_07090*	Glycosyltransferase	250,062 -> 251,189	synthesis	1128
	Hypothetical protein	*rfbX, QX99_01555*	RfbX protein (membrane)	251,287 -> 252,771	other	1485

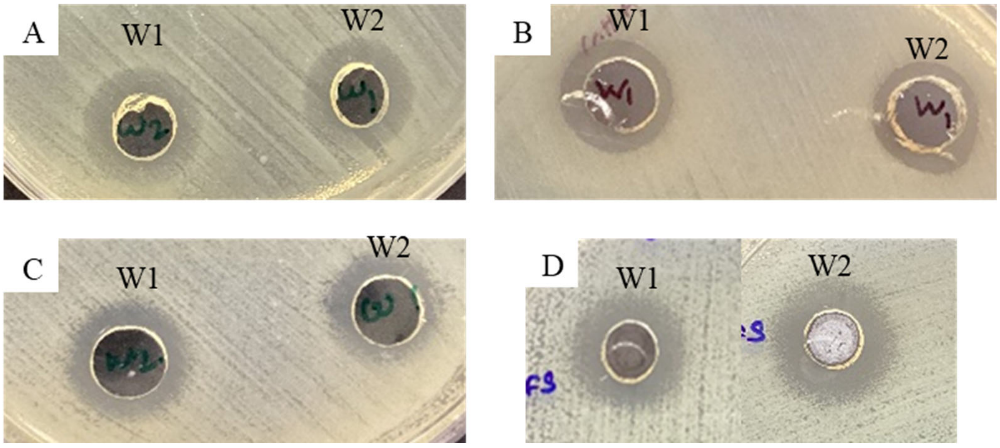

Figure S2. Antibacterial activity of CFS of *W. confusa* W1 and W2 strains against (A) *Salmonella. enterica* serotype Typhi DMST 22842 (B) *Vibrio cholerae* DMST 2873 (C) Carbapenem-resistant *Acinetobacter baumanii* SK005 (D) *Stenotrophomonas maltophilia* DMST 19079 using agar-well diffusion method (Tests are done three independent times)
